# Biogenic silver nanoparticles (AgNPs) from *Tinosporacordifolia* leaves: An effective antibiofilm agent against *Staphylococcus aureus* ATCC 23235

**DOI:** 10.3389/fchem.2023.1118454

**Published:** 2023-03-07

**Authors:** Sreejita Ghosh, Somdutta Mondol, Dibyajit Lahiri, Moupriya Nag, Tanmay Sarkar, Siddhartha Pati, Soumya Pandit, Abdullah A. Alarfaj, Mohamad Faiz Mohd Amin, Hisham Atan Edinur, Muhammad Rajaei Ahmad Mohd Zain, Rina Rani Ray

**Affiliations:** ^1^ Department of Biotechnology, MaulanaAbulKalam Azad University of Technology, Kolkata, West Bengal, India; ^2^ Department of Biotechnology, University of Engineering and Management, Kolkata, West Bengal, India; ^3^ Department of Food Processing Technology, Malda Polytechnic, West Bengal State Council of Technical Education, Govt. of West Bengal, Malda, India; ^4^ Skills innovation and Academic network (SIAN) Institute-ABC, Balasore, Odisha, India; ^5^ NatNov Private Limited, Greater Noida, Odisha, India; ^6^ Department of Life Science, Sharda University, Noida, India; ^7^ Department of Botany and Microbiology, College of Science, King Saud University, Riyadh, Saudi Arabia; ^8^ Environmental Technology Division, School of Industrial Technology, UniversitiSains Malaysia, Penang, Malaysia; ^9^ Renewable Biomass Transformation Cluster, School of Industrial Technology, UniversitiSains Malaysia, Penang, Malaysia; ^10^ School of Health Sciences, UniversitiSains Malaysia, Health Campus, Kelantan, Malaysia

**Keywords:** Tinospora cordifolia, phytoextract, green synthesis, biogenic silver nanoparticles, biofilms, *Staphylococcus aureus*, biofilm

## Abstract

Medicinal plants are long known for their therapeutic applications. *Tinospora cordifolia* (commonly called gulancha or heart-leaved moonseed plant), a herbaceous creeper widely has been found to have antimicrobial, anti-inflammatory, anti-diabetic, and anti-cancer properties. However, there remains a dearth of reports regarding its antibiofilm activities. In the present study, the anti-biofilm activities of phytoextractof *T. cordifolia* and the silver nanoparticles made from this phytoextract were tested against the biofilm of *S.taphylococcus aureus*, one of the major nosocomial infection-producing bacteria taking tetracycline antibiotic as control. Both phytoextract from the leaves of *T. cordifolia*, and the biogenic AgNPs from the leaf extract of *T. cordifolia*, were found successful in reducing the biofilm of *Staphylococcus aureus*. The biogenic AgNPs formed were characterized by UV- Vis spectroscopy, Field emission Scanning Electron Microscopy (FE- SEM), and Dynamic light scattering (DLS) technique. FE- SEM images showed that the AgNPs were of size ranging between 30 and 50 nm and were stable in nature, as depicted by the zeta potential analyzer. MIC values for phytoextract and AgNPs were found to be 180 mg/mL and 150 μg/mL against *S. aureus*respectively*.* The antibiofilm properties of the AgNPs and phytoextract were analyzed using the CV assay and MTT assay for determining the reduction of biofilms. Reduction in viability count and revival of the *S. aureus* ATCC 23235 biofilm cells were analyzed followed by the enfeeblement of the EPS matrix to quantify the reduction in the contents of carbohydrates, proteins and eDNA. The SEM analyses clearly indicated that although the phytoextracts could destroy the biofilm network of *S. aureus*cells yet the biogenicallysynthesizedAgNPs were more effective in biofilm disruption. Fourier Transformed Infrared Radiations (FT- IR) analyses revealed that the AgNPs could bring about more exopolysaccharide (EPS) destruction in comparison to the phytoextract. The antibiofilm activities of AgNPs made from the phytoextract were found to be much more effective than the non-conjugated phytoextract, indicating the future prospect of using such particles for combatting biofilm-mediated infections caused by *S aureus.*

## 1 Introduction

Development of biofilm is a natural tendency of bacteria, to survive adversities (Donlan, 2002). It is a syntrophic cluster of sessile bacterial cells, remaining shielded by aself-secreted extracellular polymeric substance (EPS). With the help of such polymeric substances, the bacterial cells can spread over biotic and abiotic surfaces and their binding with such surfaces plays a significant role in spreading the pathogenicity and virulence of the respective bacteria (Haiko and Westerlund-Wikström, 2013). The diversity of the biochemical constitution of the EPS matrix leads to different capacities of surface adherence with the substratum, which in turn causes a wide range of adaptations to adverse environmental conditions (Kostakioti et al., 2013). The EPS prevents the penetrance of antibiotics and other drugs resulting in the development of antibiotic resistance and multi-drug resistance. In today’s clinical point of view, biofilm-associated chronic and acute infections are the most challenging to treat, and day by day these infections start to threaten human health globally *Staphylococcus aureus*is one of the predominant bacteria responsible for causing community-acquired and nosocomial infections and such infections often become life-threatening (Silva- Santana et al., 2020). *S. aurues*is a Gram-positive bacterium and it is a human commensal that continuously colonizes the frontal niches of about 20%–25% of fit adult communities and 60% remain occasionally colonized (Ellis et al., 2014). The attachment of *S. aureus*to the surfaces of medical implants and host tissues results in the development of matured biofilm resulting in the persistence of chronic infections (Foster et al., 2014). The development of biofilm and their residence in the EPS matrix lessens their susceptibilities to different antibiotics and host immunity, which makes these infections difficult to eradicate (Chatterjee et al., 2014). The biofilms of *Staphylococcus aurues* are known to cause infections such as bacteremia, endocarditis, multiple sclerosis and sepsis (Sheykhsaran et al., 2022). Various parameters lead to the development of biofilms. These factors include particular gene expressions and communication among proteins that help in adherence of the biofilm to the substrate. During more advanced stages of the infections caused by *Staphylococcus aureus* biofilms, bacterial cells get dispersed from the biofilms and get spread to the secondary sites thereby worsening the infection. The World Health Organization (WHO) has classified a list of antibiotic-resistant pathogens, based on the priority of finding new alternative therapies for treating those infections, which can no longer be treated with the available antibiotic therapeutic strategies. There are three stages of priority list pathogens: Priority 1 (critical), priority 2 (high) and priority 3 (medium). Among the priority list of pathogens, WHO has considered *S. aureus* in the second priority group (high priority) because *S. aureus* is resistant against a wide class of antibiotics including methicillin and vancomycin. Vancomycin was one of the last resort antibiotics to eradicate the methicillin-resistant *S. aureus* (MRSA) infections. Prolonged or suboptimal exposure to vancomycin has led to the development of decreased vulnerability of the *S. aureus* associated infections. This decreased susceptibility can be also due to the thickening of the EPS matrix and various antibiotic inactivating enzymes present in the EPS matrix (Al-Marzoqi et al., 2020).

Silver is an antimicrobial substance with antiseptic, antibacterial, and anti-inflammatory properties (Fong et al., 2005). When silver is in a soluble state, such as Ag^+1^ or Ag^0^ clusters, it is physiologically active. Ag+ is the ionic version of silver in silver sulfadiazine, silver nitrate, as well as other ionic silver compounds. Silver is used as an ingredient in various ointments, creams, medicines, and medical instruments. Nanoparticles of silver have unique characteristics attributed to their very small size, which helps in enhancing their bioavailability and efficacy. Silver nanoparticles (AgNPs) possess inhibitory actions on bacterial, fungal, and viral growth (Jeong et al., 2005). AgNPs are increasingly explored by researchers because of their cytotoxic and antibacterial potential due to their easy attachment with the bacterial cell walls. This adherence impacts the cellular respiration and permeability leading to cell death. Moreover, AgNPs can easily enter the cells and bind with the biomolecules, including protein and DNA through their phosphorous and sulfur groups, respectively (Aabed and Mohammed, 2021). Besides, their antimicrobial properties, their biosynthesis process is comparatively cost-effective (Capek, 2004). Physical and chemical methods of AgNPs synthesis involve the use of toxic reagents and result in a very low yield of AgNPs. However, the biogenic method of AgNPs synthesis is environment-friendly, readily-scalable, simple and involves only natural reagents, so they are free from potential toxicities and also biocompatible (Emeka et al., 2014). Hence, the biological methods for the preparation of nanoparticles have more advantages over chemical and physical methods of AgNP synthesis, *via* processes like ultrathin film procedure, thermal evaporation, synthesis through diffusion flame, lithographic process, electrodeposition, sol-gel technique, chemical solution and vapor deposition, catalytic process, hydrolysis and method of co-precipitation (Hu et al., 2020). However, the exact mechanism of anti-biofilm activities of AgNPs is not yet clearly understood. It is being presumed that their extremely small sizes lead to the increase in oxidative stress within the bacterial cells through the generation of reactive oxygen species (ROS) or through denaturation of the fatty acids present inside the cell membrane and increasing peroxidation of lipids. Once, the AgNPs penetrate the cells they destabilize the intracellular biomolecules and structures leading to the death of the bacterial cells (Rozhin et al., 2021). Table 1 below describes about the potential applications of AgNPs in different fields.

**Table:** 

Field of application	Utility of the AgNPs in the field	References
Anaesthetics	Breathing mask coating and coating of endotracheal tubes for mechanical support of ventilation	Mocanu et al. (2013)
Diagnostics	Pyramids made of nanosilver to enhance the process of biological detection. Ultrafast and ultrasensitive platforms for clinical diagnosis of myocardial infarction. Fluorescence associated sensing of RNA with turntable coated with AgNPs having plasmonic properties	Lee and Jun (2019)
Drug therapy	Remote controlled LASER light associated microcapsule opening	Ivanova et al. (2018)
Optics	Coating of contact lens	Nguyen et al. (2021)
Imaging	Dendrimer and silver nanoconjugate to label cells. Silver sand nanoconjugates as fluorescent core shell nanoballs used in molecular and cellular imaging of the malignant cells	Homan et al. (2010)
Neurology	Catheter coating for drainage of cerebrospinal fluid (CSF)	Wallace et al. (2017)
Orthopaedics	Bone cement additive used in implantable substances manufactured with layers of clay andAgNP stabilized with starch. Intramedullary nail coating used in fractures of long bones. Implant coating in orthopaedic stockings for replacement of joints.	Poon et al. (2021)
Patient care	AgNP used as superabsorbent nanogel for incontinence substances	Shen et al. (2018)
Pharmaceutical	Dermatitis treatment. AgNPs inhibit the replication of HIV-I, helps in treating ulcerative colitis, wound healing characteristics	Mathur et al. (2018)
Biomedical	Anti-fungal, anti-bacteria, anti-inflammatory, anti-viral, anti-cancer and anti-angiogenic agent	Burdusel et al. (2018)
Textiles	Medicinal devices and textiles, textile coating to block UV rays	Mirzaei et al. (2021)
Food industries	Food processing and packaging using AgNP coatings	Zorraquin- Pena et al. (2020)
Environmental applications	Water and air disinfection, disinfection of groundwater, drinking water and biological wastewater	Jaffri and Ahmad (2018)

**TABLE 1:** GC-MS analysis of phytoextract of *T. cordifilia*

Sl No	Rt	Name of the compounds	Molecular formula	Molecular Wt (g/mol)	Structure
1	4.94	Isopinocarveol	C_10_H_16_O	152.23	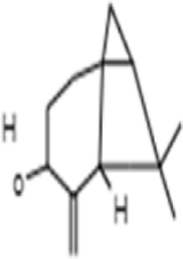
2	8.60	Caryophyllene	C_15_H_24_	204.35	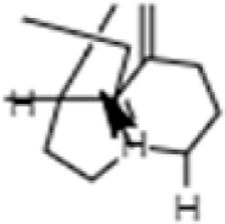
3	9.38	Benzene, 1-(1,5-dimethyl-4-hexenyl)-4-methyl	C_15_H_22_	202.34	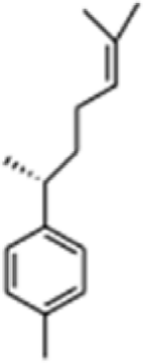
4	10.45	(+)-Sativen	C_15_H_24_	204.35	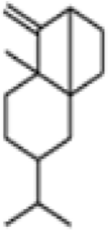
5	10.78	Methyl 4,7,10,13-hexadecatetraenoate	C_17_H_26_O_2_	262.4	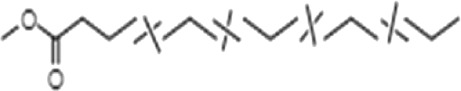
6	11.70	7-epi-cis-sesquisabinene hydrate	C_15_H_26_O	222.37	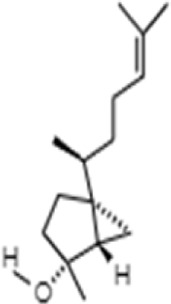
7	12.13	2,5-Octadecadiynoic acid, methyl ester	C_19_H_30_O_2_	290.4	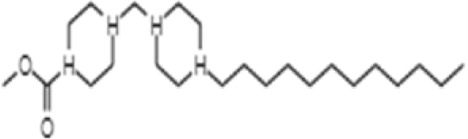
8	12.71	Phenol, 2-methyl-5-(1,2,2-trimethylcyclopentyl)-, (S)-	C_15_H_22_O	218.33	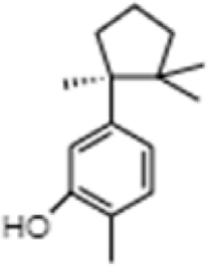
9	13.33	Phenol, 2,4-bis(1,1-dimethylethyl)	C_22_H_30_O	310	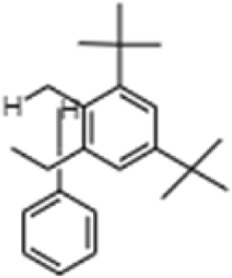
10	14.97	5-Isopropyl-2,8-dimethyl-9- oxatricyclo [4.4.0.0 (2,8)]decan-7-one	C_14_H_22_O_2_	222.32	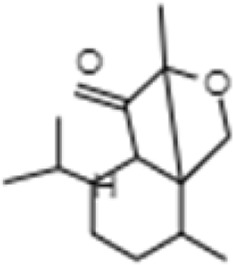
11	17.79	17-Octadecynoic acid	C_18_H_32_O_2_	280.4	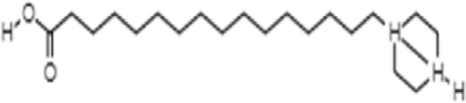
12	17.96	Z,Z,Z-4,6,9-Nonadecatriene	C_19_H_34_	262.5	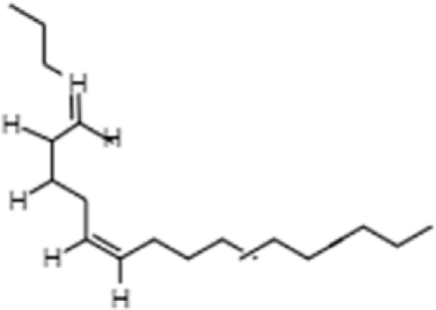
13	20.51	n-Propyl cinnamate	C_12_H_14_O_2_	190.24	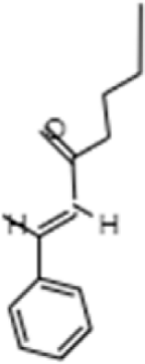
14	26.71	Dasycarpidan-1-methanol, acetate (ester)	C_20_H_26_N_2_O_2_	326.4	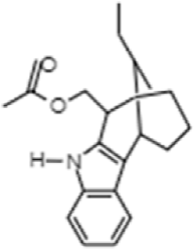
15	28.86	Piperine	C_17_H_19_NO_3_	285.34	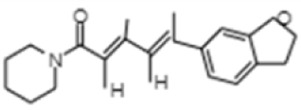

In this study, we threw light on the green synthesis of AgNP from the leaf extracts of *T. cordifolia*and tested the efficacies of the phytoextract alone as well as biogenic AgNPs as antibiofilm agents against the biofilms formed by *S. aureus.* This study also investigated the antibacterial properties of the AgNP nanoconjugates biogenically synthesized from *T. cordifolia* leaf extract along with investigation of anti-biofilm activity. The leaf extracts of *T. cordifolia* are generally enriched with diterpenoids, sterols, aliphatics and alkaloids. These bioactive compounds can potentially act as reducing agents in the biosynthesis of AgNPs. Pertinent to the increased resistance against the available antibiotics, AgNP nanoconjugates have been synthesized to overcome the problem of antibiotic resistance as well as to punctuate the persistence of acute and chronic biofilm-associated infections caused by *S. aureus* since biofilms are extremely impermeable to the penetration of antibiotics or other antibacterial agents due to the presence of multiple drug efflux pumps and antibiotic inactivating enzymes. The nanoconjugates of silver synthesized from the leaf extract of *T. cordifolia* can penetrate deeper and reach the sessile bacterial cells residing within the biofilms of *S. aureus* due to their small sizes.

## 2 Materials and methods

### 2.1 Preparation of *Tinospora cordifolia* leaf extract

The leaves of *T. cordifolia*were collected from the local gardens of West Bengal under the guidance of Botanist, India and they were washed with double distilled water and dried. After that, the leaves were crushed with 95% methanol followed by incubation for 16–24 h at room temperature (Quave et al., 2012). Then the phytoextract was sieved using a gauge cloth followed by centrifugation at 5000 rpm for 10 min and the supernatant was collected and stored at 4°C for further use. The methanol used was of analytical grade and purchased from HiMedia.

#### 2.1.1 Synthesis of biogenic AgNP preparation using leaf extract of *Tinospora cordifolia*

10% w/v of properly washed and dried *T. cordifolia*leaves were crushed in 20 mL of double distilled water. The aqueous extract was then sieved with a gauge cloth followed by centrifugation at 5,000 rpm for 10 min and the supernatant was collected. 90 mL of 1 mM silver nitrate solution was prepared, to which, 10 mL of phytoextract was added dropwise while stirring with a magnetic stirrer (Jalal et al., 2016). The biosynthesis of AgNPs was confirmed by the change in color from white to reddish brown. This reaction mixture was further centrifuged at 5000 rpm for 10 min and the pellets were collected followed by washing twice with distilled water and re-suspended in phosphate buffered saline (PBS).

### 2.2 Characterization of the biologically synthesized AgNPs

The biogenically synthesized AgNPs were characterized using various techniques such as UV-Vis spectroscopy, Field Emission Scanning Electron Micrograph (FE- SEM), Dynamic Light Scattering (DLS) and measurement of zeta potential, which are all listed below.

#### 2.2.1 Characterization using UV-Vis spectroscopy

The synthesis and stability of biogenic AgNPs can be detected by the UV-Visible spectra of the AgNP solution (Sharma et al., 2020). Double distilled water was used as blank. The absorbance spectra of the reddish brown AgNP solution were recorded at wavelengths ranging from 300 to 700 nm by using Lasany LI- 294/296 Microprocessor single beam UV-Vis spectrophotometer.

#### 2.2.2 Characterization by using Field Emission Scanning Electron Microscope (FE- SEM)

The biogenically synthesized AgNPs were dropped cast on a cover slip and oven-dried (Jalal et al., 2016). Then the dried sample of AgNPs was visualized under JEOL JSM- 7600F Field Emission Scanning Electron Microscope at a voltage of 15 kV to determine the surface morphology of the biogenic AgNPs.

#### 2.2.3 Characterization by using Dynamic Light Scattering (DLS) and zeta potential measurement

The hydrodynamic diameter, zeta potential (surface charge), and particle distribution intensity (polydispersity index/PdI) of AgNPs can be examined using DLS by the process of measuring the dynamic variations of the intensity of light scattering caused by Brownian motion of the particles (Elamawi et al., 2018). All the measurements were performed in triplicate with 1min of equilibration time at 25°C temperature in a ZetasizerNanoseries (Nano- ZS). The mode of data processing was set to high multi-modal resolution.

### 2.3 Cultivation of the *Staphylococcus aureus* ATCC 23235 working strain and development of biofilm

Biofilm forming strain of *Staphylococcus aureus* ATCC 23235 was grown in Luria Bertani broth (SRL) overnight at a temperature of 37°C at pH 7.4. The bacterial strains that were used in this study were cultured within an Erlenmeyer flask of 100 mL containing 50 mL of Luria Bertani broth at pH seven and were incubated for 24 h at 37°C. The biofilm formation by the working bacterial strain was analyzed by the use of the microplate assay method. Biofilm growth depends on the synergistic activities of the concentrations of sugar and salt. The optimal concentration for the formation of biofilms was found by adding various concentrations of glucose (0.25%–10% w/v) and NaCl (0.5%–7% w/v) in the culture broth. All the experimental setups were incubated at 37°C for a period of 72 h.

### 2.4 Study of antimicrobial activities of phytoextract and biogenic AgNPs

Antimicrobial activities of phytoextract from *T. cordifolia*and biogenic AgNPs and standard antibiotics were detected by analyzing the diameter (in millimeters) of the inhibition zones obtained by agar well diffusion method. Various agar plates containing the test bacterial strain were treated with phytoextracts, AgNPs, and antibiotics at different concentrations in wells, punctured on the plates. The plates were incubated at 37°C for 24 h and observed for inhibition zones in accordance with the specifications made by the National Committee for Clinical Laboratory Standards (Patra et al., 2014).

### 2.5 Determination of minimum inhibitory concentration (MIC)

The MIC values of the phytoextract from *T. cordifolia*and the biogenic AgNPs were determined against *S. aureus* ATCC 23235 by the technique of microdilution (Jeyaseelan and Jashothan, 2012). 10 µL of *S. aureus* ATCC 23235 was inoculated in 5 mL of LB broth and treated with phytoextract (concentrations ranging from 10 to 50 μg/mL) and biogenic AgNPs (concentrations ranging between 1 and 50 μg/mL), except in the control tubes. The test tubes were incubated at 37°C for a period of 24 h and the bacterial growth intensity was measured spectrophotometrically at 660 nm.

### 2.6 Determination of minimum biofilm eradication concentration

The minimum biofilm eradication concentration was calculated using an MTT assay (Baishya et al., 2016). In 96-well plates, added100 µl of LB broth and 2 µL of *S. aureus* ATCC 23235 was added to each well and incubated at 37°C for 72 h. The LB broth was discarded after 72 h at 37°C to remove the planktonic cells, and 20 µL of phytoextract and biogenic AgNPs was added to all of the wells except the control (untreated microbe) followed by the addition of MTT reagent and mixing it well with the help of cyclomixer including control and incubated at 37°C for 4 h and the absorbance was measured at 550 nm using Thermo Scientific Multiskan Sky ELISA plate reader.

### 2.7 Determination of reduction of biofilm formation by *Staphylococcus aureus* ATCC 23235 on treatment with phytoextract and biogenic AgNPs

The microtiter plate technique was used to estimate biofilm formation quantitatively (Lahiri et al., 2021b). In this method, six-well plates were used and each well was filled with 5 mL LB Broth a single coverslip was dropped in each of the wells, and 20 µL of *S. aureus* ATCC 23235 cells was added to all the wells. After 72 h of incubation at 37°C, 100 µL of treatment was added and kept in the incubator for 24 h at 37°C. The planktonic cells were gently removed and the coverslip was taken out and stained with crystal violet dye (0.1% w/v in acetic acid) for 1 min. After discarding the excess crystal violet dye, the stained cells were treated with acetic acid for 1 min and absorbance of the acetic acid from the coverslip was measured at 540 nm spectrophotometrically. A sterile growing medium only and a functioning solution were employed as negative and positive controls, respectively, in the assay. The following formula was used to compute the percentage of biofilm inhibition:

$$Percentage\;\left(\%\right)\;of\;biofilm\;inhibition=\left(OD\;at\;540\;of\;non-treated\;cells\right)-\frac{\left(OD\;at\;540nm\;of\;treated\;cells\right)}{\left(OD\;at\;540nm\;of\;non-treated\;cells\right)}X\;100$$ (1)

### 2.8 Detection of viability and revival count of the sessile bacterial cells

The bacterial cells were grown in an LB broth containing chitin flakes (0.1% w/v) for a period of 72 h and then the broth was discarded to remove the planktonic cells and the chitin flakes were washed with sterile double distilled water. Followed by washing, fresh LB broth was added followed by the addition of 240 µL of phytoextract, AgNPs, and standard antibiotic (tetracycline). The bacterial growth was spectrophotometrically determined at 660 nm at regular time intervals of 2 h (Ding et al., 2015). After measuring the viability count of the sessile biofilm cells, the remaining broth from the test tubes was discarded and the sessile cells, which were attached to the chitin flakes were washed with sterilized double distilled water to eliminate any planktonic cells before being refilling with 5 mL of fresh LB broth in each test tube. The density of these cells was spectrophotometrically measured at 660 nm to detect any revival of the biofilm-forming cells after withdrawing the treatment.

### 2.9 Enfeeblement of extracellular polymeric substances (EPS)

The mechanism of extraction of EPS associated with the biofilm-forming bacterial cells, 30 µL of *S. aureus* ATCC 23235 inoculum was added to 15 mL of LB broth containing chitin flakes and grown for a period of 72 h at a temperature of 37°C. After 72 h, the LB broth was discarded to eliminate the planktonic cells and the chitin flakes were washed with sterile double-distilled water. Then 240 µL of phytoextract and AgNPs were added to the respective conical flasks, except for the control, and incubated for a period of 1 h. 5 mL of phosphate-buffered saline (PBS) at pH 7.2 was added in each conical flask and cyclospinned for 2–3 min for breakage of the biofilm by hindering the interactions and keeping the EPS components together within the matrix. The PBS was then transferred to 15 mL flacon tubes followed by centrifugation at 6000rpm for 15 min s at 4°C. The pellet was re-suspended in 2.5 mL of 10 mM TrisHCl (at pH 7.8). 20mM beta-mercaptoethanol (BME) and 1 mM phenylmethylsulfonyl fluoride (PMSF) was added to the above suspension in a ratio of 1:1. The cell suspension was provided with heat shock by placing it between hot water and ice for 5 min each and the process was repeated for 4–5 times for all the samples. The suspensions were then centrifuged at 5000 rpm for 30 min at 4°C and the supernatant was transferred in fresh tubes followed by the addition of 1 mL of 10% Trichloroacetic acid (TCA) in acetone and incubated for at least 72 h at 4°C. After 72 h, the suspensions were again centrifuged at 5000 rpm for 30 min at 4°C followed by the washing of the protein pellet with 90% acetone and air drying. The pellet was dissolved in 500 µL of rehydration buffer (Teanpaisan et al., 2017).

#### 2.9.1 Reduction in total carbohydrate content of EPS

The content of carbohydrates present in the EPS was quantified using the Anthrone method (Meade et al., 1982). In this method, to each of the EPS samples, 50 µL of 80% phenol was added and mixed completely for 2 mins using vortex followed by the addition of 2 mL of concentrated sulfuric acid and the color turned to deep red. The mixture was incubated at room temperature for 10 min before being measured spectrophotometrically for a reduction in carbohydrate content in EPS at 490 nm.

#### 2.9.2 Reduction in total protein content of EPS

Protein content was estimated by using Bradford assay, which is the shortest sensitive method of simple dye binding assay and was developed by Marion M. Bradford in 1976. In this method, 2 µL of samples were loaded in a 96-well plate with 100 µL of Bradford reagent followed by incubation at room temperature for 5 min, and the absorbance of protein content in the EPS was measured using Thermo Scientific Multiskan Sky ELISA plate reader at 595 nm.

#### 2.9.3 Reduction in total eDNA content of EPS

For estimating the eDNA content of EPS, the EPS samples were diluted with cold 100% ethanol in a 1:3 ratio followed by incubation at 4°C for 2 h and spectrophotometrically measured at a wavelength of 560 nm.

### 2.10 FTIR analysis of the EPS matrix after treatment

Biofilm produced by *S. aureus* ATCC 23235 on chitin flakes in LB broth for 72 h at 37°C was treated separately with phytoextract of *T. cordifolia* and the biogenic AgNPs followed by drying in a hot air oven. The FT-IR spectra were recorded in the range between 450 and 4000 cm^-1^ with a PerkinElmer FT- IR Spectrometer (Frontier) (Pati et al., 2020).

### 2.11 Detection of biofilm reduction by scanning electron microscopy (SEM)

Biofilms developed on chitin flakes in LB broth after incubation for 72 h at 37°C, were treated with the phytoextract as well as with the biogenic AgNPs and incubated for 2 h at 37°C. Thereafter, the broth was discarded and the chitin flakes were washed with 0.9% (w/v) NaCl for removing any leftover planktonic cells. The samples were then suspended in 2.5% glutaraldehyde for 20 mins followed by repeated dehydration using upgraded ethanol. The dried chitin flakes containing the sessile colonies were visualized under a ZEISS EVO- MA 10 scanning electron microscope (Lahiri et al., 2021a).

### 2.12 Reagents and chemicals

All chemicals and reagents used in the experimentation are of analytical grade and were purchased from HiMedia and SRL.

### 2.13 Statistical analyses

All the experiments were performed in triplicate and the results were depicted as mean ± SD (standard deviation).

## 3 Results and discussion

### 3.1 Identification of the bioactive compounds from the phytoextract of *Tinospora cordifolia*

It was observed that the phytoextract of *T. cordifolia* comprised of different chromophoric groups such as -OH, phenolic, unsaturated carbonyls, *etc.* Mass spectrum interpretation through GC-MS of unidentified bioactive compounds and comparing them with the database stored in National Institute Standard and Technology (NIST) library verified the biochemical identity of 15 compounds from *T. cordifolia* extract*.* The molecular formula, name of the compounds, peak area, molecular weight, and bioactivity of the experimental materials were determined. The relative percentage composition of each bioactive compound was calculated by comparison with the average peak area with respect to the total area (Table 1).

### 3.2 Characterization of the biogenic AgNPs

#### 3.2.1 Determination of UV- Vis spectra of the biogenically synthesized AgNPs

**FIGURE 1:**
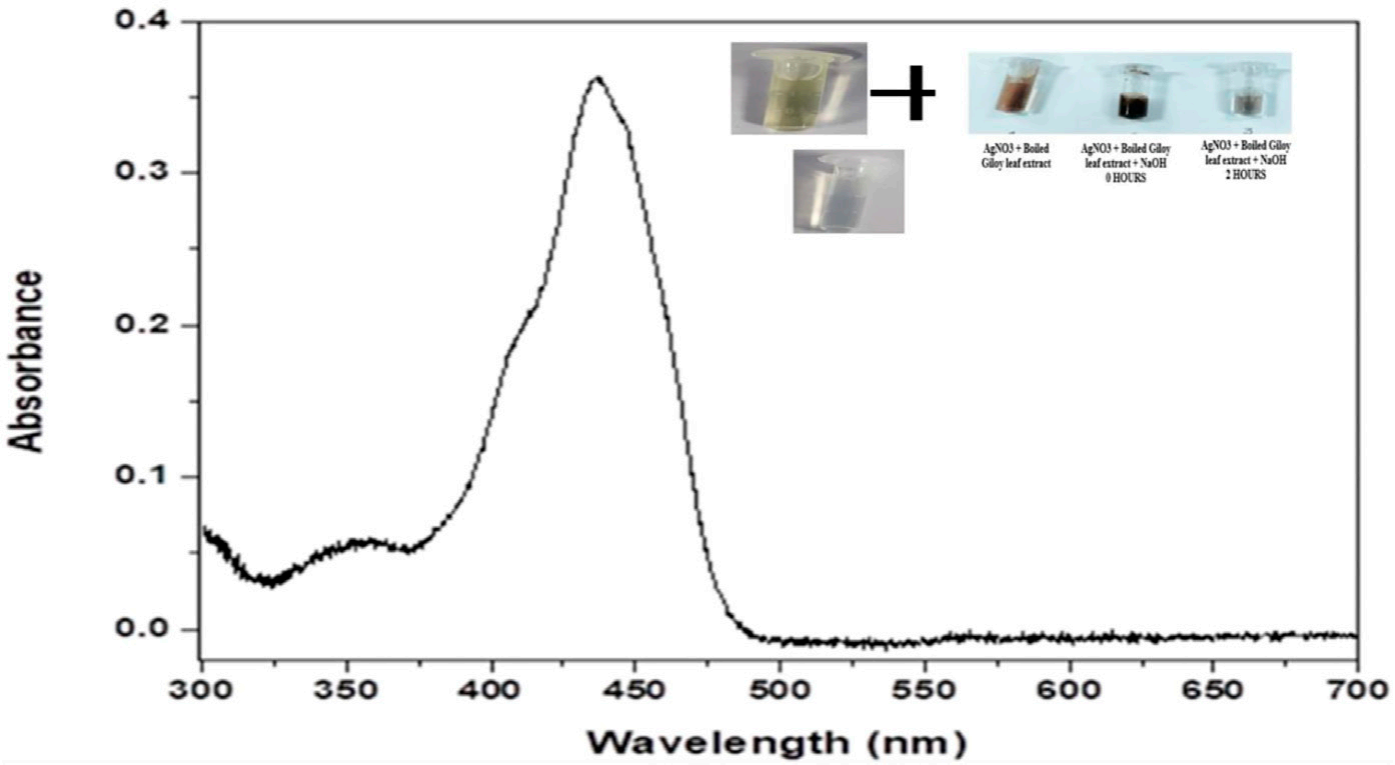
Graphical representation UV- Visible absorption spectra of AgNPs from leaf extract of *Tinospora cordifolia* showing absorbance peak at the wavelength of 430 nm.

UV-Vis spectroscopy acts as an important technique for the purpose to detect the synthesis of AgNPs with the monitoring of electronic structures and optical properties of the synthesized NPs. The electron clouds undergo oscillation on the surface of the NPs possessing the ability to absorb the electromagnetic waves possessing a particular frequency. This mechanism is termed surface plasmon resonance (SPR) which in turn is being recorded by the use of a UV-Vis spectrophotometer (Smitha et al., 2008). The UV- Visible spectra of the biogenically synthesized AgNPs using the leaf extract of *T. cordifolia* presented a peak at 430 nm (Figure 1) in correspondence with the surface plasmon response of AgNPs. The peak was similar to the work performed by Marhaby and Seoudi 2016 which also depicted the AgNPs synthesized by 4-Nitrophenol fetched at a peak at 423 nm (Almarhaby and Seoudi, 2016). Another work showed the peak of biogenic AgNPs from *Clinacanthus nutans* at 450 nm (Mat Yusuf et al., 2020). Biogenic synthesis of AgNPs is based on conditions like the type of solvent being used, the reducing agent, and the non-toxic substance being used for the purpose of stabilizing the nanoparticles (Raveendran et al., 2003). The change in the intensity of the wavelength is based on the increase in the number of NPs that are formed as a result of the reduction of silver ions along with the biomolecules that are present within the system. It is usually observed that the SPR bands become sharper and undergoes a shift to shorter wavelengths with the rise in temperature indicating a decrease in the size of the particles. The reduction in the size of the NPs is due to an enhancement in the reduction time during the mechanism of synthesis. Consumption of silver ions takes place during the process thereby blocking the phenomenon of secondary reduction taking place on the surface of AgNPs (Yang and Li, 2013). It has been observed that NPs that absorb wavelengths between 400 and 900 nm are spherical in shape (Yusuf et al., 2020).

## 4 Particle size distribution and surface charge analysis of the biogenic AgNPs

**FIGURE 2:**
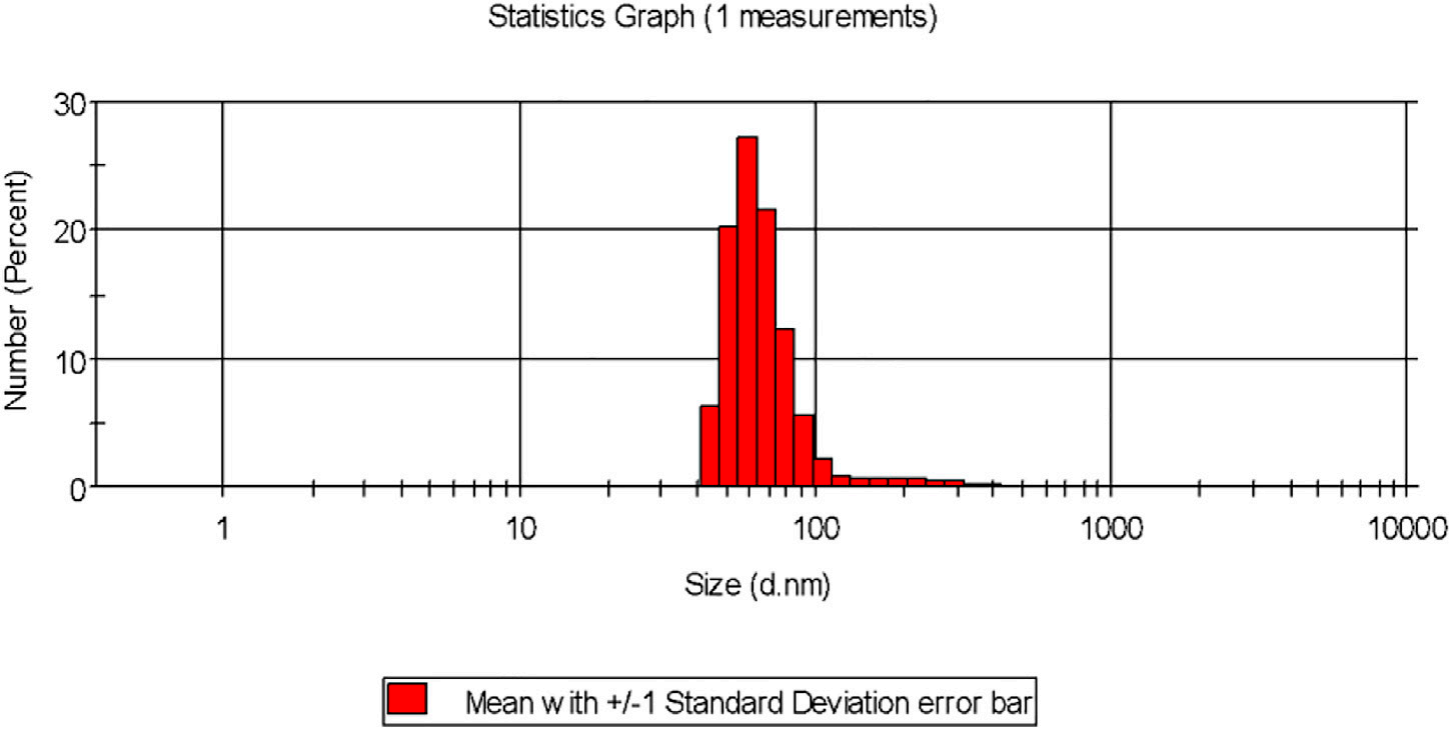
Particle size distribution of the biogenic AgNP solution synthesized from the leaf extract of *Tinospora cordifolia*.

Dynamic light scattering (DLS) is the technique used for the purpose of measuring the average size of the NPs within liquid suspension requiring fewer volumes of samples. The measurement of the size is based on the Brownian motion theory which denotes the random movement of the particles randomly in suspension or gas. The dynamic fluctuation from the intensity of light scattering is used for measuring the average size of the NPs (Murdock et al., 2008). The size of the biogenically synthesized AgNPs ranged between 43.82 ± 1.023 nm- 91.28 ± 1.12 nm (Figure 2). A considerable number of peaks appeared below 100 nm (Table 2).

**TABLE 2:** Size of the particle and zeta Potential analysis.

Type of NPs	Polydispersity index (PDI)	Size of the particle (nm)	Zeta potential (mV)
Green-synthesized AgNPs	0.201 ± 0.005	43.82 ± 1.02–91.28 ± 1.12	−11.37 mV

**FIGURE 3:**
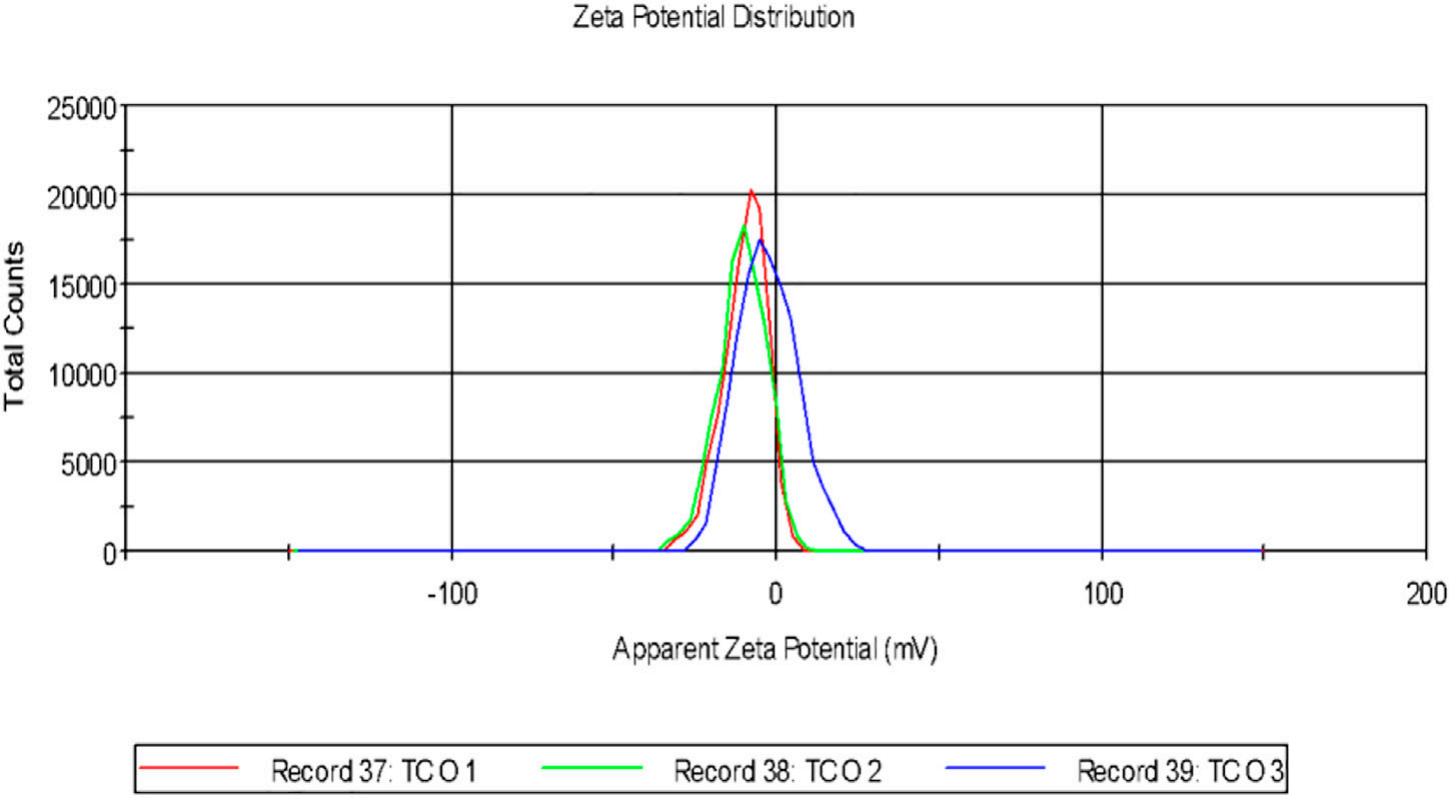
Zeta potential of AgNPs biosynthesized from the leaf extract of *Tinospora cordifolia*.

It was observed that the calculated PDI was 0.201 ± 0.005 for the green-synthesized NPs which is within the range from 0–1 in which 0 signifies monodisperse and one is polydisperse (Murdock et al., 2008). Thus the result signifies that the synthesized AgNPs were present in the monodisperse phase and aggregations of particles were minimum. Experimental conditions have a direct influence on morphology, size, and stability (Ghorbani et al., 2011). The agglomeration of NPs sometimes occurs due to the presence of bioactive compounds present within the solution (Shameli et al., 2012). The charges of the moving particles under the impact of the electric field can be calculated with the help of Zeta Potential (Bhattacharjee, 2016). Negative charges were observed around the particle which does not represent the actual surface charge (Figure 3). The presence of the negative charge is due to the absorption of bioactive compounds on the surface of AgNPs(Moldovan et al., 2016). Temperature plays a vital role in the regulation of the stability of the NPs and thereby increases the value of zeta potential. The high amount of zeta potential results in the development of repulsive forces thereby preventing aggregation of the particles (Priyadarshini et al., 2013).

### 4.1 FE- SEM analyses of the AgNPs

**FIGURE 4:**
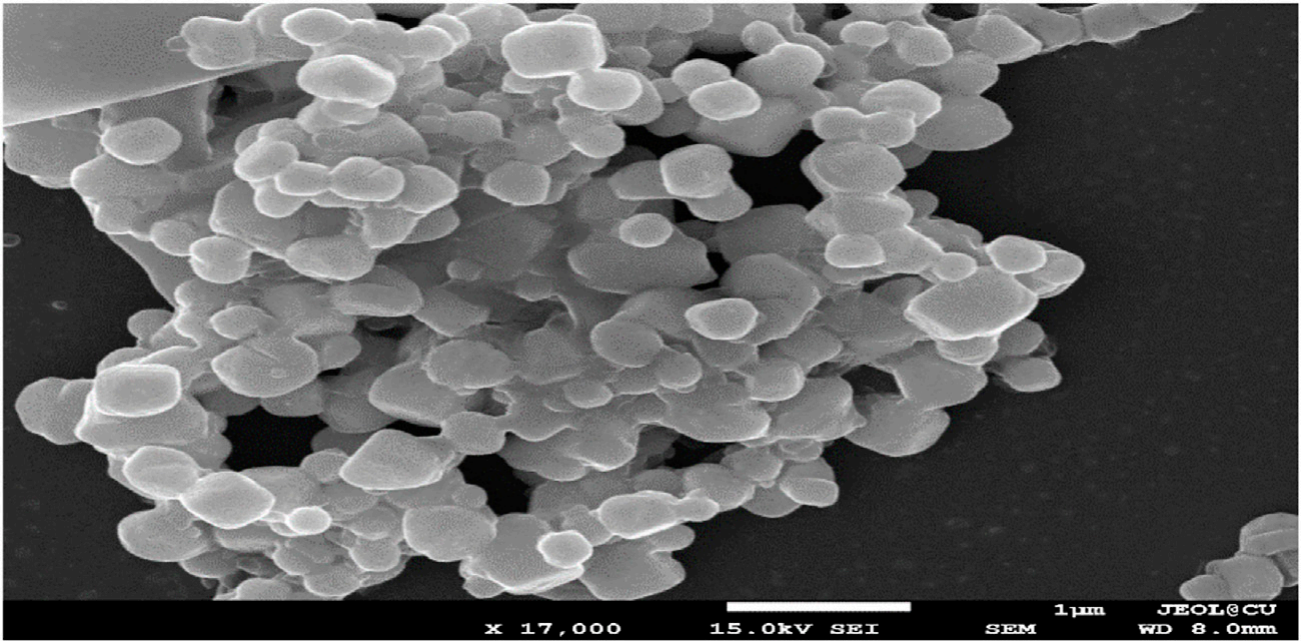
FE- SEM images of biogenic AgNPs from *T. cordifolia*leaves.

Green-synthesized AgNPs were studied under FE-SEM and it was observed that the NPs were spherical in shape (Figure 4) that was as per the SPR peak being observed in the UV-spectroscopy. The peak was observed at 430 nm which indicated the spherical nature of the NPs. In the presence of a protective agent, the sides of the NPs showed slightly elliptical or oval (Ahila et al., 2016), High surface tension and energy resulted in the agglomeration of the NPs (Wang et al., 2020).

### 4.2 Antimicrobial activity determination of the phytoextract and AgNPs against *Staphylococcus aureus* ATCC 23235

**FIGURE 5:**
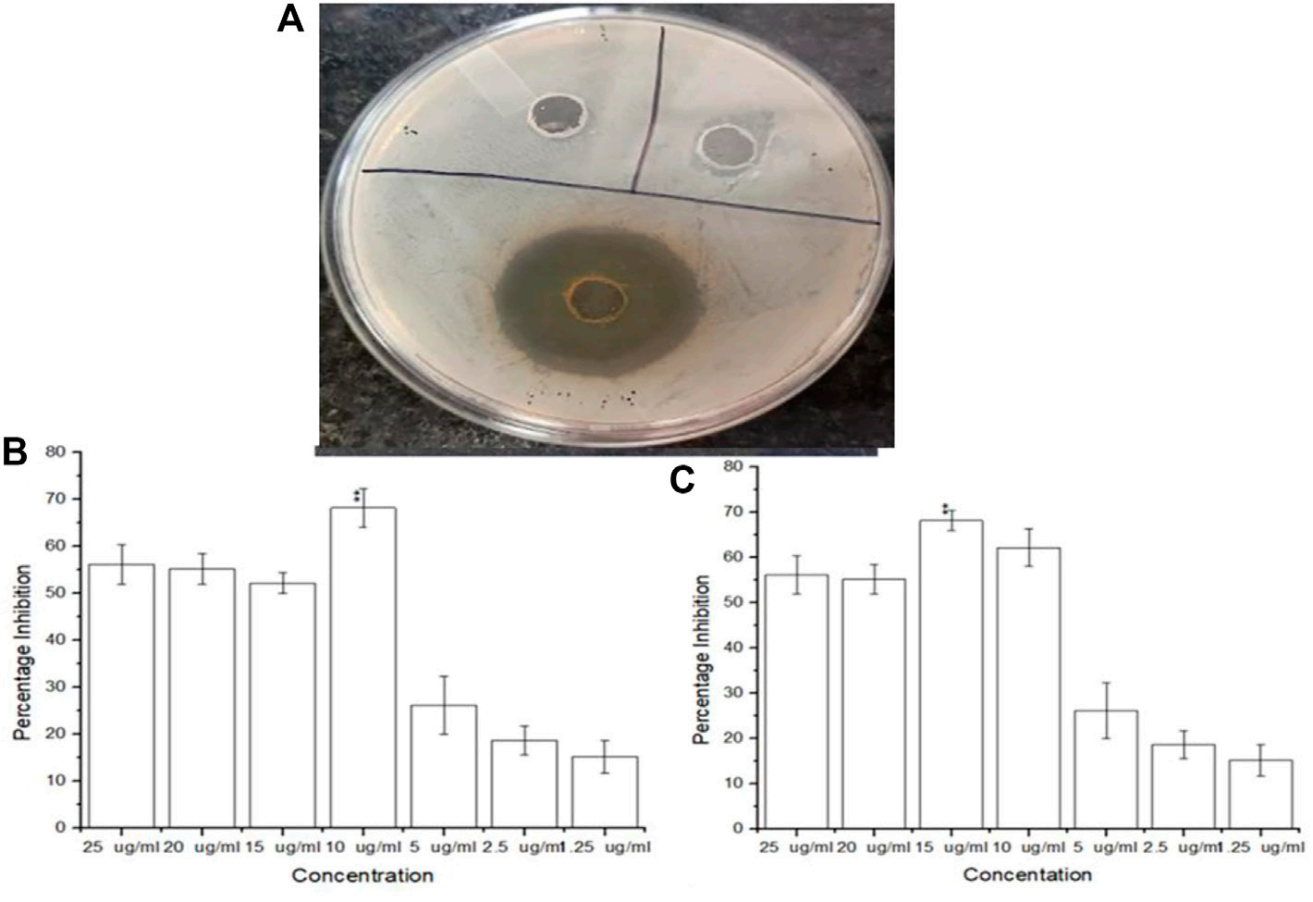
**(A)** Inhibitory zones on addition of AgNPs **(B)** Antimicrobial efficacy of biogenic AgNPs from *Tinospora cordifolia* and **(C)** Antimicrobial efficacy of the phytoextract of *Tinospora cordifolia*.

Amongst the phytoextract and the biogenic AgNPs, the biogenic AgNPs depicted zones of inhibition of 12–18 mm while that of the phytoextract was 10–15 mm (Figure 5A). A control setup was arranged using ethanol and tetracycline that did not show significant antimicrobial activity against *S. aureus* ATCC 23235 proving that the test bacteria developed resistance against ethanol as well as a tetracycline antibiotic. The biogenic AgNPs possessed a MIC value of as low as 10 μg/mL (Figure 5B) while the phytoextract showed a MIC value of 15 μg/mL (Figure 5C), which is quite higher in comparison to the AgNPs. This determines that the phytoextract along with AgNPs can have better effects against *Staphylococcus aurues* ATCC 23235 than phytoextract alone. This may be due to the higher penetration capacities of the AgNPs, which can penetrate deep within the bacterial cells and bring about their destruction. This observation was similar to the previously published work where the AgNPs exhibit an inhibitory effect within the range of 4–64 μg/mL (Attallah et al., 2022).

### 4.3 Antibiofilm property determination of phytoextract and AgNPs

**FIGURE 6:**
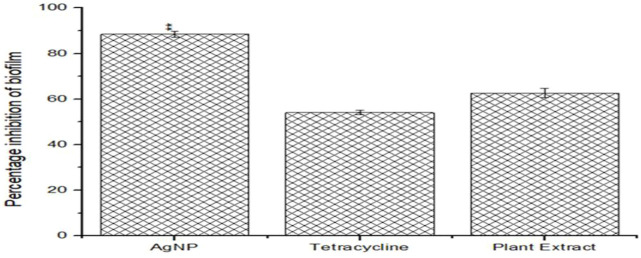
Anti-biofilm efficacies of biogenic AgNPs, phytoextract and antibiotic Tetracycline against *Staphylococcus aureus* ATCC 23235.

Both the phytoextract as well as the biogenic AgNPs demonstrated antibiofilm properties against *S. aureus* ATCC 23235 (Figure 6). However, the biogenic AgNPs showed a percentage reduction of more than 83.14% ± 0.56% of the biofilm, while that of the phytoextract and tetracycline were around 60.12% ± 1.23% and 50.25% ± 0.87% respectively. The reduction of biofilm by AgNPs were found to be statistically significant (*p* < 0.01) Figure 1. The biosynthesized AgNPs exhibited greater efficacy of action due to the doped bioactive compounds from the plant source (Miškovská et al., 2022).

### 4.4 Reduction in viability and revival count of the sessile cells after treatment

**FIGURE 7:**
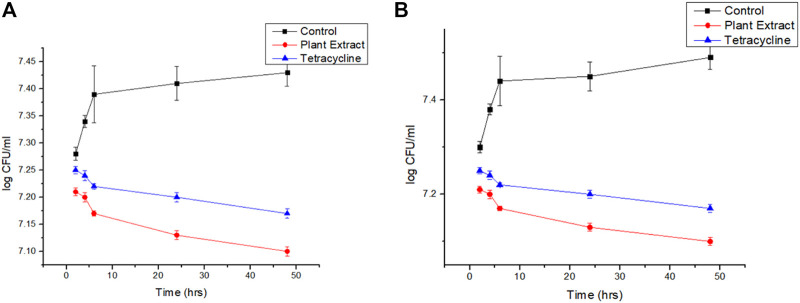
**(A)**: Viability count reduction followed by treatment with phytoextract, AgNPs, and tetracycline and **(B)** Revival of the cells after withdrawal of the treatment.

It was found that the viability count of the sessile colonies of *Staphylococcus aurues* ATCC 23235 demonstrated the highest reduction in the presence of biogenic AgNP than the phytoextract and tetracycline (Figure 7A). The efficacy of the biofilm eradication was further validated by the negligible revival of the cells after the withdrawal of the respective treatments for 24 h (Figure 7B).

### 4.5 Reduction in the contents of the biofilm matrices of *Staphylococcus aureus* ATCC 23235 by biogenic AgNPs and phytoextract

**FIGURE 8:**
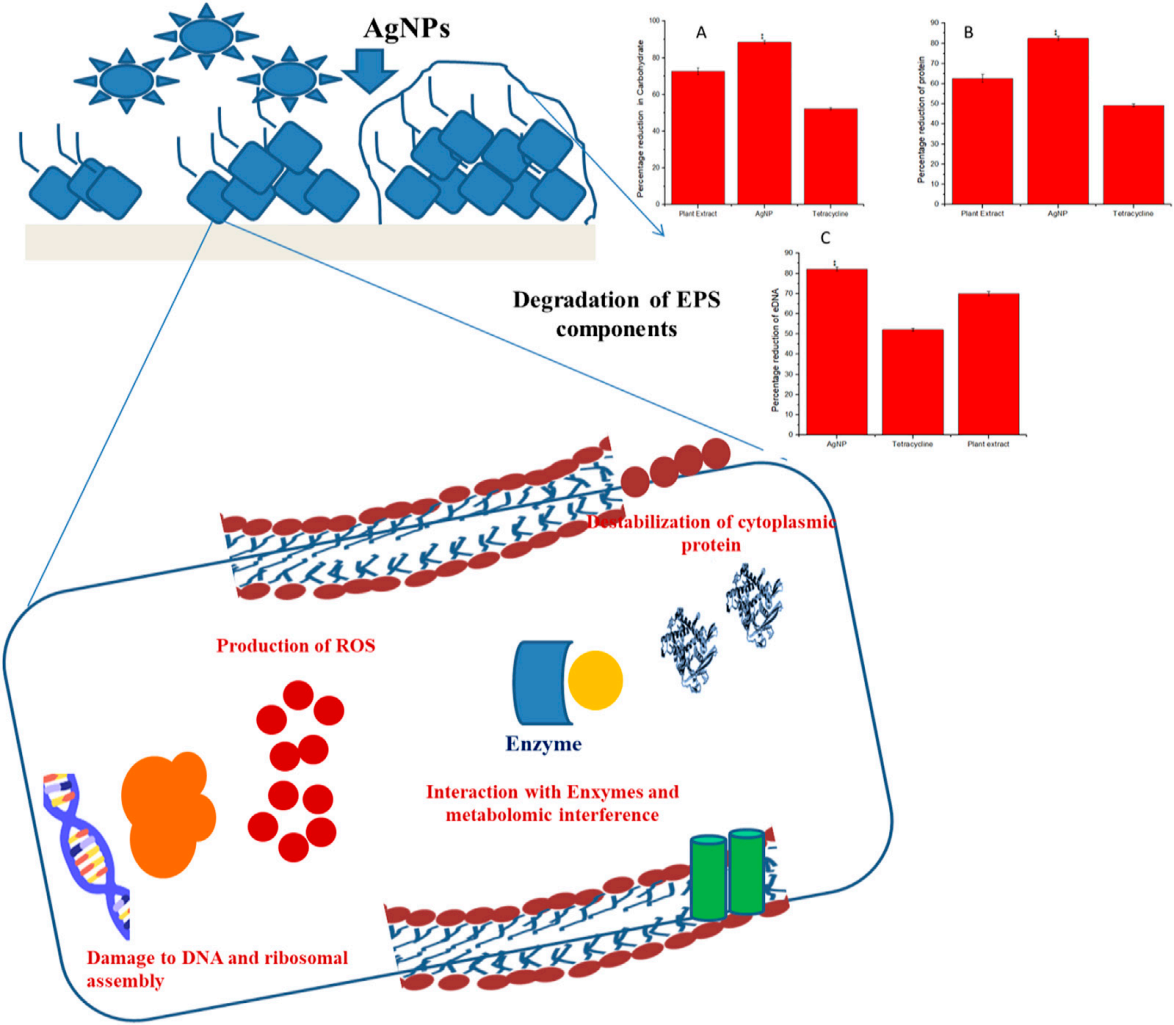
Probable mode of action involved in the eradication of biofilm matrix by biogenically synthesized AgNPs from *Tinospora cordifolia*.

Structural composition disruption of the biofilm matrices, namely carbohydrates, proteins, and eDNA leads to biofilm destabilization. It was observed that the maximum reduction of carbohydrates, proteins, and eDNA of the EPS matrix was brought about due to the activity of the biogenically synthesized AgNPs than the phytoextract alone or tetracycline. Carbohydrate content was reduced by about 90.12% ± 0.56% by the biogenic AgNPs while that with phytoextract and tetracycline were about 65% ± 1.03% and 50% ± 0.98% (Figure 8A). The content of protein was significantly reduced by around 85% ± 1.26% by the biogenic AgNPs while that with phytoextract and tetracycline were around 65% ± 0.75% and 45% ± 1.02% (Figure 8B). The eDNA content was reduced to about 80% ± 1.23% by the biogenic AgNPs in comparison to the phytoextract, which reduced the eDNA content by 65% ± 0.85%, and tetracycline, which decreased the content of eDNA by 50% ± 0.45% (Figure 8C).

### 4.6 FTIR analysis of the EPS modification by the biogenic AgNPs and phytoextract

**FIGURE 9:**
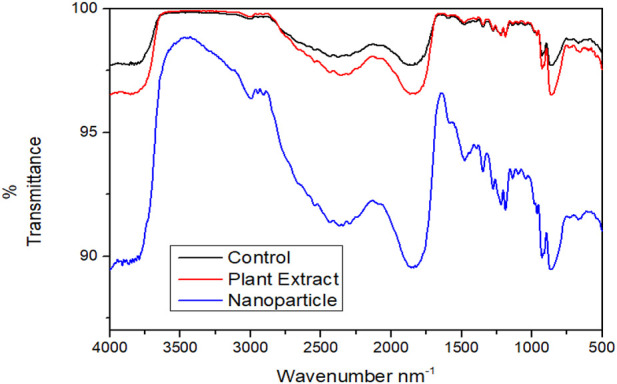
Comparative FT-IR spectra showing the effect of treatment by phytocompound and AgNPnanoconjugate.

FT- IR was performed for analyzing the modifications in the functional groups of the EPS matrix of *S. aureus* ATCC 23235 after treating with the biogenically synthesized AgNPs and the phytoextract (Figure 9). Remarkable modifications in the spectral regions of polysaccharides (890–1175 cm^-1^), lipids (3,000–2800 cm^-1^), proteins (1700–1500 cm^-1^), and nucleic acids (1,300–900 cm^-1^) were analyzed in the FT- IR spectroscopy. The biogenic AgNPs brought about the highest reduction in peak intensities, shape alterations, and shifts in wavelengths of *S. aureus* ATCC 23235 in comparison with the control sample and sample treated with phytoextract. This suggests that the biogenic AgNPs could directly interact and decrease the concentration of various EPS constituents such as polysaccharides, lipids, and nucleic acids as evidenced by the reduced peak intensity data.

### 4.7 Photomicrographic analyses of the removal of biofilm by treatment with biogenic AgNPs and phytoextract

**FIGURE 10:**
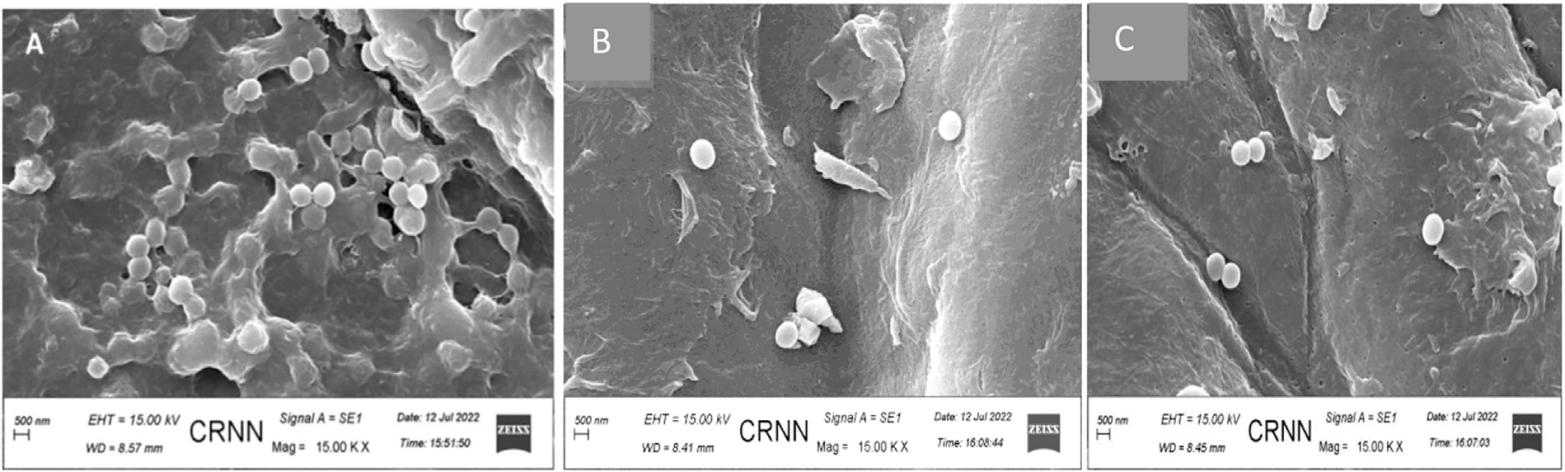
Scanning electron micrograph of *Staphylococcus aureus* **(A)** before and **(B)** after treatment with biogenic AgNPs **(C)** *Staphylococcus aureus* ATCC 23235 after treatment with phytoextract seen at a magnification of × 15000.

The best anti-biofilm activity was observed with the biogenic AgNPs than the phytoextract and this indicates that the phytoextract acts synergistically well when combined with NPs than phytoextract alone. AgNP- treated (Figure 10B) and phytoextract-treated (Figure 10C) bacterial sessile cells were observed under SEM, which depicted clear biofilm disruption of the sessile cells after treatment as compared with the control samples (Figure 10A).

## 5 Conclusion

The application of natural products for human welfare is time immemorial and their usage are getting enhanced with every passing day. *Tinospora cordifolia* is a widely available weed possessing numerous health-beneficial activities (Ahmad et al., 2021) and can be used successfully for irreversible disruption of the biofilm-associated cells of *S. aureus*. *T. cordifolia*can also be effectively used in the green synthesis of biogenic AgNPs from silver nitrate. Such kind of NP synthesis can be deemed to be environmentally friendly since it is free from any type of harmful chemicals or reducing substances since the entire NP synthesis process is biogenic. However, the exact mode of action of AgNPs on bacterial cells is yet to be known in detail. Some of the experimental results indicated that these NPs mainly interact with the cell surfaces of several bacteria (Mikhailova, 2020). On the surfaces of cells, the AgNPs get adhered to the cell wall and cell membrane of bacteria thereby penetrating deep inside the intracellular organelles and modifying the biomolecular signal transduction pathways. In the case of Gram-positive bacteria such as *S. aureus*, the AgNPs find their way to the cytoplasm by membrane property modification leading to the dissipation of proton motive force (PMF) and lead to the damage in the bacterial cell due to membrane destruction (Durán et al., 2016). The penetration of AgNPs lead to the development of oxidative stress within the cells leading to the generation of reactive oxygen species (ROS), which oxidize the double bonds of the membrane fatty acids allowing the production of free radicals and damage to the cell membrane (Vega-Baudrit et al., 2019). The preliminary step for the formation of biofilms gets inhibited by the presence of AgNPs. This is because the AgNPs can bind with the cellular surface thereby altering the adhesive compounds such as extra polymeric matrices, which are involved in the aggregation of bacterial cells and biofilm formation (Gurunathan et al., 2014).

The biologically synthesized AgNPs have demonstrated good anti-biofilm efficacy, suggesting that they could be employed as an antibiofilm weapon against the biofilm-associated infections caused by *S. aureus*. Experimental observations clearly indicated that biofilm removal is accomplished through irreversible denaturation of EPS matrices and subsequent inhibition of biofilm formation by *S. aureus*. The mode of action of these biogenic AgNPs synthesized from the leaf extract of *T. cordifolia*is mainly by EPS matrix denaturation. Hence, these NPs can act as potential drug candidates for controlling chronic and persistent infections caused by the biofilms of *S. aureus*.

## Data Availability

The original contributions presented in the study are included in the article/supplementary material, further inquiries can be directed to the corresponding authors.
